# A Traceable Vaccine Supply Management System

**DOI:** 10.3390/s22249670

**Published:** 2022-12-10

**Authors:** Yaohong Ai, Chin-Ling Chen, Wei Weng, Mao-Lun Chiang, Yong-Yuan Deng, Zi-Yi Lim

**Affiliations:** 1 School of Computer and Information Engineering, Xiamen University of Technology, Xiamen 361024, China; 2 School of Information Engineering, Changchun Sci-Tech University, Changchun 130600, China; 3 Department of Computer Science and Information Engineering, Chaoyang University of Technology, Taichung 41349, Taiwan; 4 Bachelor Degree Program of Artificial Intelligence, National Taichung University of Science and Technology, Taichung 40401, Taiwan; 5 Department of Information and Communication Engineering, Chaoyang University of Technology, Taichung 41349, Taiwan

**Keywords:** blockchain, vaccine safety, EdDSA, supply chain, transparency

## Abstract

Everyone should be vaccinated, but the eligibility and safety of the vaccine are always overlooked by most people. The outbreak of COVID-19 has led many countries to intensify the development and production of the COVID-19 vaccine. and some countries have even required universal vaccination against this epidemic. However, such popularization of vaccination has also exposed various flaws in vaccine management that existed in the past, and vaccinators have become more concerned about the effectiveness of their vaccinations. In this paper, we propose a blockchain-based traceable vaccine management system. First, the system uses smart contracts to store the records generated during the whole process, from vaccine production to vaccination. Second, the proposed scheme uses the Edwards-curve digital signature algorithm (EdDSA) to guarantee the security and integrity of these data. Third, the system participants can access the corresponding data according to their authority to ensure the transparency of the whole system operation process. Finally, this paper will also conduct a security analysis of the whole system to ensure that the system can resist potential attacks by criminals.

## 1. Introduction

### 1.1. Background

Since ancient times, humans have been constantly battling infectious diseases. Over thousands of years, many infectious diseases have devastated human society. The Black Death that struck Europe in 1347 claimed at least 25 million fresh lives in just four years, which is almost 40% of the total population of Europe [1]. The Spanish flu of 1918 infected about a quarter of the world’s population at that time, causing about 50 million deaths [2]. The smallpox virus also caused about 400,000 deaths per year in 18th-century Europe [3]. However, from these tragic experiences, people have also come up with effective ways to combat infectious diseases. For example, the suppression of the Black Death was inseparable from the effective isolation of those infected with the Black Death by the government in the Middle Ages [4]. In addition, in 1796 the English physician Edward Jenner successfully prevented smallpox by implanting the cowpox virus into the human body; this material is also known as a vaccine [3]. Ever since then, vaccination, as an important part of modern disease control, has embodied the protection of susceptible people. The emergence of various vaccines against infectious diseases has also made vaccination the most economical and effective method of infectious disease prevention.

Nowadays, vaccination is closely related to people’s lives. Many countries have implemented free vaccination policies to reduce the likelihood of citizens contracting some common infectious diseases. On the other hand, people are generally in agreement with the act of vaccination. However, the management of vaccine-related records is still relatively backward. There are even some underdeveloped areas in the world that are still using the oldest paper form to record and save various messages about vaccines. This management of such a large number of records in physical form alone will inevitably lead to a series of huge safety risks for vaccine management. Meanwhile, the quality of vaccines in these less-developed regions will always be difficult to guarantee. Therefore, to address these problems, the World Health Organization (WHO) proposed a blueprint for improving global vaccine safety [5], emphasizing the need to safeguard vaccines in low- and middle-income countries (LMICs). In recent years, the rapid development of digital technology, such as the emergence of electronic health records (EHRs), has greatly facilitated the storage of medical data. Therefore, various types of vaccine-related records can be stored digitally in various government-run healthcare institutions. In this case, the safety of vaccines is generally ensured by governmental agencies, such as the lot release agency mentioned in the article [6], which conducts sampling surveys of vaccines, and only those vaccines that pass various quality tests can be sold. However, this way of managing vaccine information through a central institution also has many hidden dangers. For example, the failure of a single node will lead to the paralysis of the whole system [7,8], the increasing amount of various medical data will bring a huge burden to the servers of the central institution [8,9], and the central node is more vulnerable to the attacks of malicious nodes [10]. Supervision by central institutions likewise makes vaccine information highly opaque. If government regulation is not effective, vaccines that do not meet manufacturing standards will be extremely difficult to detect immediately. Once these fraudulent vaccines reach the market, they will cause great harm to the people and will bring a crisis of confidence to the government and the vaccine industry, in turn. In addition, the global outbreak of the COVID-19 pandemic in early 2020 has made several countries require universal COVID-19 vaccination regulations and issue corresponding vaccination certificates to those who have received the vaccine, and only those who hold such certificates are permitted to enter public places [11,12,13,14,15]. While recommending countries use electronic vaccination certificates, WHO formed the Smart Vaccination Certificate consortium [16,17] as a way to monitor the COVID-19 vaccination programs of individual countries and prevent the further spread of the epidemic. The traditional centralized vaccine record management approach is not only difficult to meet the demand for the frequent recall of increasingly large vaccine data but also faces serious security and privacy challenges. Therefore, the technology of vaccine record management needs some revolution. The emergence of blockchain technology will be able to bring innovation to the storage of vaccine records. Blockchain technology was first proposed by Satoshi Nakamoto in 2008 [18]. The essence of Blockchain is a distributed ledger that was initially used as the underlying technology for Bitcoin. Due to its characteristics of decentralization, transparency, security, and anonymity, blockchain technology was soon applied in various fields, among which it has been widely used in the medical field, where information is highly sensitive [19,20,21]. Blockchain also plays a pivotal role in dealing with a similar pandemic, and the European Parliament even listed blockchain technology as one of the top ten technologies that could effectively mitigate the impact of the COVID-19 epidemic [22].

In summary, a blockchain-based vaccine information management system is proposed in this paper. Our research goals are as follows:

1. The records generated from the manufacture, procurement, distribution, and vaccination up to the diagnosis of vaccine side effects and the identity information of each player will be permanently stored in the blockchain system. All information is guaranteed with integrity and security. In addition, each user in the system can trace the corresponding records based on his or her identity, ensuring the transparency of the whole system.
2. The use of Burrows-Abadi-Needham (BAN) logic ensures that two unfamiliar nodes confirm each other’s identity, ensuring the authenticity of each other’s identity and the trustworthiness of the information exchanged between nodes.
3. The system using blockchain can resist potential risks, such as replay attacks and man-in-the-middle attacks.

### 1.2. Related Works

Since Mettle [23] first proposed the application of blockchain in healthcare, numerous scholars have researched the application of blockchain technology in healthcare and have agreed that blockchain technology can make great contributions to improving the quality of healthcare services and enhancing the security of healthcare data. Gorden et al. [9] argued that all kinds of interoperability within the healthcare system would change from traditional institution-driven to patient-driven. They focus on the significant contribution that blockchain technology can make to this patient-driven model of healthcare interactions. Because of the revolution that blockchain can bring to the healthcare field, many articles have proposed corresponding blockchain-based healthcare systems. Azaria et al. [24] proposed a system called MedRec, which uses a permissioned blockchain to manage electronic medical records (EMRs). MedRec protects the security and integrity of EMRs while making them traceable by specific roles through authorization, and the ledgers generated during the operation of the system will be audited in case of disputes. However, the system is only a prototype without a working model, and the local storage of huge amounts of medical data is not reasonable. However, the scheme has no working model, and the local storage of massive amounts of medical data is not reasonable. Zhang et al. [20] developed a blockchain-based app named FHIRChain. The app normalizes and stores clinical data in Fast Healthcare Interoperability Resources (FHIR) standards. To solve the problem of clinical data silos, FHIRChain uses public keys to represent the identity of app users, thus ensuring that these standardized clinical data can be securely shared among authenticated medical personnel. Moreover, the article diagrams the components of the app and the user registration and authorization processes. However, the scheme does not perform a security analysis. Kumar et al. [25] designed a medical data-sharing system using Hyperledger Fabric. Each transaction in the system will be protected by an identity-based broadcast group signcryption scheme (IDBGSC). After passing the Practical Byzantine Fault Tolerance (PBFT) consensus protocol, the transaction information will be written into the ledger, which ensures that the security and integrity of these highly private medical data are guaranteed. The proposed system is then evaluated for security and performance to demonstrate its practical value. However, when the data in the system are disputed, it is not possible to quickly locate the actual signed individual users in the group. Kumar et al. [26] used federated chains and IPFS technology to manage patient information in a distributed manner. The system can easily store huge and cumbersome medical information under the chain, while the chain order stores the content that addresses the hashes of the files. This not only improves the system throughput but also makes the authorization of private information more convenient. However, the article has fewer parts for information security analysis and does not mention how to prevent some common attacks.

However, in contrast to the high enthusiasm of people studying how to use blockchain technology to secure healthcare data, researchers are less likely to think about how the technology can be used to secure vaccine-related information [27,28]. Sigwart et al. [27] suggested the possibility of applying blockchain technology to the vaccine supply chain, but a specific architectural part is missing. Yong et al. [28] proposed a system based on blockchain technology and machine learning to secure vaccines. Each vaccine has an exclusive Radio Frequency Identification (RFID) to prevent the possibility of vaccine information being falsified. The vaccine-related information recorded in the system can also be traced by consumers and regulators, solving problems such as vaccine expiration or vaccine information forgery. However, the article lacks an analysis of potential attacks.

Nevertheless, since the worldwide outbreak of the COVID-19 epidemic in 2020, many countries have had considerable requirements for vaccination and the checking of vaccination certificates against the pandemic [11,12,13,14,15,16,17]. These works on how to use blockchain to ensure the security of the vaccine and its related information also launched a fierce discussion [29,30,31,32,33]. Ricci et al. [29] referred to the feasibility of applying blockchain technology in the transport of the COVID-19 vaccine and proof of COVID-19 vaccination. Deka et al. [30] proposed a method to maintain individual vaccination records and proof of immunization by introducing blockchain technology. Then, the vaccination records and proofs stored in the system are managed through IPFS. However, the content lacks a detailed description of the entire vaccine data management process. Antal et al. [31] used smart contracts to monitor vaccine distribution and vaccination. Every vaccine has its corresponding batch number, and temperature changes are constantly monitored by temperature sensors during the storage and transportation of the vaccine. Vaccine recipients can also trace the lot identification of the vaccine they received and access vaccine-related information after vaccination. Chauhan et al. [32] used smart contracts to implement four aspects of the system: registration of each role, monitoring of the vaccine distribution process, tamper-proofing of vaccine information, and vaccination feedback. The registration of each role will hash the private key of the role with its address and generate a unique QR code based on this hash value. Users can access the system based on their QR codes. However, both of the above articles are missing part of the security analysis. Chen et al. [33] proposed a blockchain-based vaccine record storage system. Although the system has a more complete system architecture and security analysis, the system has a complete framework and security analysis. The security of the elliptic curve digital signature algorithm (ECDSA) is not strongly guaranteed, and the proposed method involves the vaccination phase.

The remaining sections of this article are organized as follows. Section 2 briefly introduces the techniques mentioned in the article. Section 3 describes the details of the system design in detail. Section 4 provides a security analysis of the proposed scheme. Section 5 then analyzes the performance of the system. Finally, Section 6 concludes the paper to some extent.

## 2. Preliminary

### 2.1. Smart Contract

The concept of a smart contract was first introduced by Nick Szoba [34] and is essentially a computer program or transaction protocol that can be executed spontaneously. A smart contract can reduce the involvement of third-party intermediaries. As long as the participants reach an agreement with each other, the smart contract can be executed spontaneously according to the protocol. With the emergence of Ethereum [35], smart contracts have often been used in blockchains. Because of the feature that smart contracts can be executed spontaneously as long as the conditions are met, they reduce the possible omissions caused by manual operations in the system and also improve the operational efficiency of the blockchain system. In addition, because smart contracts do not require the participation of third-party trust institutions, the security and privacy of data are further ensured. On the other hand, smart contracts do not require the participation of third-party trust institutions, and the security and privacy of data are further ensured. 

### 2.2. EdDSA

The Edwards-curve digital signature algorithm (EdDSA) was proposed by Bernstein et al. [36] in 2012. EdDSA uses a variant of the Schnorr signature based on twisted Edwards curves [37]. It has high performance across platforms while ensuring high security. Crucially, the random number value of the EdDSA is taken concerning the private key of the node and the content of the message sent, which overcomes the random number quality problem present in the ECDSA and the digital signature algorithm (DSA). Sony Corporation has caused a large number of cracks in Play Station 3 due to the random number quality problem of ECDSA [38].

### 2.3. BAN Logic

Burrows-Abadi-Needham (BAN) logic is a rule proposed by Burrows et al. [39] in 1990 for defining and analyzing message exchange protocols. This helps the user determine that the information exchanged is trusted and that the process of exchanging information is without eavesdropping by third parties. To apply BAN logic, it is necessary to first transform the messages in the protocol into formulas in BAN logic and then make reasonable assumptions based on the specific situation.

### 2.4. Security Requirements

A system is always exposed to many risks, such as attacks by criminals and data leakage, so it is essential to analyze the potential risks. The vaccine management system proposed in this paper faces the following potential risks:

1. Mutual authentication: The exchange of data is necessary for the operation of the system. To guarantee the security and privacy of the exchanged information, both parties need to authenticate the identity of the other node.
2. Integrity: The integrity of the data exchanged throughout the vaccine management system should be ensured to prevent possible data tampering and loss.
3. High-quality random number: The system needs to generate high-quality random numbers to ensure that the digital signature is not easily forged, thereby ensuring the security of the whole system.
4. Non-repudiation: Each node should not deny its actions and send messages.
5. Man-in-the-Middle Attacks: Illegal third-party nodes intercept and obtain the messages being exchanged between two communicating parties in some way.
6. Replay attack: The attacker pretends to be a legitimate message sender and sends a message to the receiving node that it has received. This process can easily cause the disclosure of node identity information.
7. Sybil attack: Sybil attack is an online network security system threat in which an attacker attempts to control a network by creating multiple fake account identities, multiple nodes, or computer coordinates.

## 3. Method

In this section, some specific details of the system implementation will be covered. The first thing that needs to be discussed is the system architecture of the system. Some notations of the system will also be listed below. 

### 3.1. System Architecture

**Figure 1:**
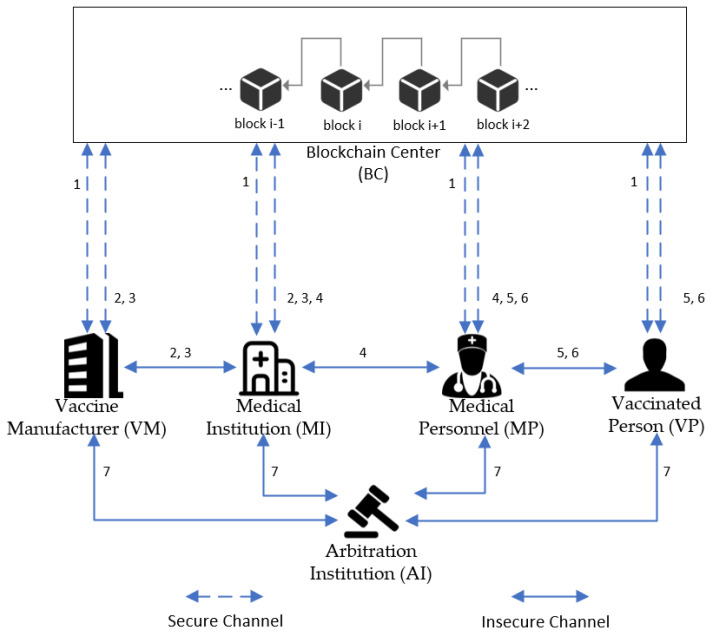
System architecture diagram.

The study proposes a blockchain-based record storage and sharing system for vaccines from production to vaccination. Figure 1 shows the main architecture of the system, which consists of six actors: the blockchain center, vaccine manufacturer, medical institution, medical personnel, vaccinated person, and arbitration institution. The detailed description is as follows.

1. Blockchain Center (BC): The blockchain center keeps most of the important information during the operation of the whole system. The registration of all nodes and the generation of public and private key pairs are done by this role. The mutual authentication between nodes will also be realized through the blockchain.
2. Vaccine Manufacturer (VM): A vaccine manufacturer is generally a third-party company that is qualified to manufacture vaccines. The vaccine is produced according to the vaccine procurement requirements of the medical institution. The vaccine manufacturer distributes the vaccine to the appropriate medical institutions in agreement with the medical institution. Moreover, it has regulatory responsibility for the transportation process.
3. Medical Institution (MI): Medical institutions purchase vaccines according to the targets given by the government. Upon receipt of the vaccine from the vaccine manufacturer, the medical institution is required to confirm the eligibility of the vaccine and store the vaccine. When the vaccine is about to be used, the medical institution needs to distribute the vaccine to the medical staff responsible for the vaccination.
4. Medical Personnel (MP): Medical personnel must be employed at the appropriate medical institution and have medical vaccination capabilities. After receiving the vaccines to be vaccinated on the day, medical personnel need to inoculate the vaccinated person.
5. Vaccinated Person (VP): Vaccinated persons are the group of people who are currently suitable for vaccination. Before vaccination, medical personnel will determine whether the vaccinated persons are eligible for vaccination by scanning the QR code. If vaccinated persons have some adverse reactions after vaccination, they will be required to submit details of the adverse reactions for further diagnosis.
6. Arbitration Institution (AI): In the case of a medical dispute that is difficult to reconcile, the arbitration institution will make a corresponding decision.

Figure 1 shows the scenario of the proposed scheme, which contains the business processes of user registration, vaccine procurement, vaccine distribution, vaccination, and side effect description. The details are as follows:

Step 1 Each role needs to register through the BC and obtain its corresponding public and private key pairs.
Step 2 The MI submits a vaccine request to a confirmed VM. Upon receiving the request, the VM verifies the identity of the MI. After confirming that the identity is correct, the VM starts to produce the vaccine and uploads the relevant data (vaccine lot number, vaccine manufacturer id, vaccine shelf life, etc.).
Step 3 Once the vaccine is made, the VM uploads the vaccine information to the BC and transports the vaccine to the corresponding MI. The vaccine transportation process requires strict compliance with transportation rules, such as the storage temperature range for each vaccine and the transport time requirements. After receiving the vaccine, the MI needs to verify that the vaccine information is correct. All related records need to be uploaded to the blockchain.
Step 4 MI needs to store vaccines after receiving them. Storage rules include a temperature between 2 °C and 8 °C, storage time cannot be longer than the remaining shelf life of the vaccine, etc. The information generated during storage needs to be uploaded to the BC. Then, the MI distributes the vaccine to be administered to qualified MP. After receiving the vaccine, the MP needs to confirm that the records related to the vaccine are accurate. Finally, the records in the blockchain are updated again.
Step 5 The VP goes to the vaccination site and submits his or her personal information and vaccination status to the MP before the vaccination. Vaccination can be carried out only after the MP has verified the information of the VP and confirmed that the VP is suitable for vaccination. After a vaccination is finished, the MP is required to update the vaccination information of the VP. The VP confirms that the vaccination information is correct. Then, the relevant records in BC will be updated.
Step 6 If a VP experiences side effects after receiving the vaccine, the VP must first provide the MP with his or her personal information, vaccination status, and details of the adverse reaction. After verifying the identity of the VP, the MP determines whether the adverse reaction is a vaccination side effect. If the adverse reaction is confirmed to be caused by the vaccination, the relevant records are uploaded to the BC. Meanwhile, the VP will receive further treatment.
Step 7 In the case of irreconcilable disputes throughout the system process, the arbitration department will obtain information from various parties for adjudication.

### 3.2. Notation

The notation of the proposed scheme is shown below:
$ID_{X}$The identity of X
$Cert_{X}$The certificate of X
pA k-bit prime number$F\left(p\right)$Finite group p
EThe elliptic curve defined on finite group p
GA generating point based on E
bAn integer b with $2^{b-1}>p$
$h_{i}$The ith bit of the hash valuenAn integer n with 3≤n≤b
$\left(d_{X},Q_{X}\right)$The EdDSA private key and public key of X
$\left(R_{X},S_{X}\right)$The EdDSA signature of X
$\left(pk_{X},sk_{X}\right)$The public key and private key of X
$M_{X-Y}$The message from  X  to  Y
$Enc_{pk_{X}}\left(M\right)$Encrypted the message M with the public key of X
$Dec_{sk_{X}}\left(M\right)$Decrypted the message M with the private key of X
H()One-way hash function$r_{X}$The random value of X based on E
$T_{X}$Timestamp message of X
△TThe threshold for checking the validity of timestamps$A\overset{?}{=}B$Verify whether *A* is equal to *B*

### 3.3. Initial Phase

**Figure 2:**
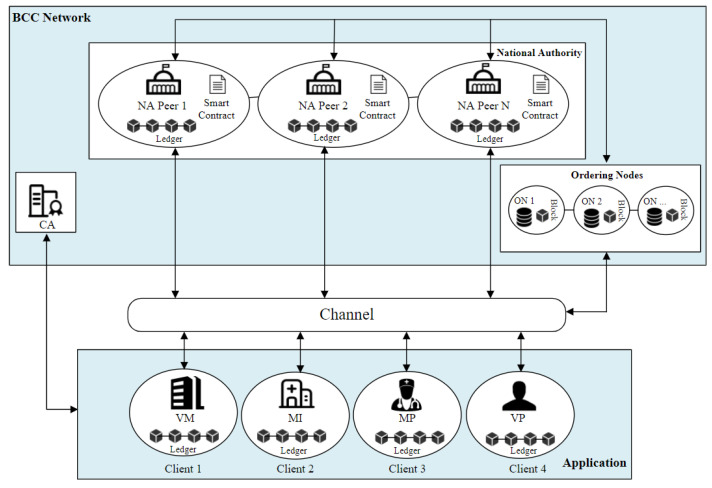
The architecture of the hyperledger fabric.

In this phase, we deployed a scalable blockchain center network based on the architecture of the Hyperledger Fabric, shown in Figure 2. The National Authority (NA) peer represents a peer controlled by government agencies, such as the Food and Drug Administration (FDA). These peers have the highest permission to use the system. The BCC also includes the certificate authority (CA), which is also generally authorized by the government to provide services to other access clients, such as VM, MI, and MP. The CA will give these access nodes the unique ID value, the public and private key pairs, and the certificate after they complete registration. The CA is also responsible for the renewal and revocation of these messages. Finally, BCC also includes ordering nodes (ON). The ON receives transactions containing signed and endorsed proposal responses from applications via a gateway service, and orders and packages the transactions into blocks. These ordered transactions are sent to peers for validation. When the consensus mechanism is passed, the peer commits the block to its ledger.

**Figure 3:**
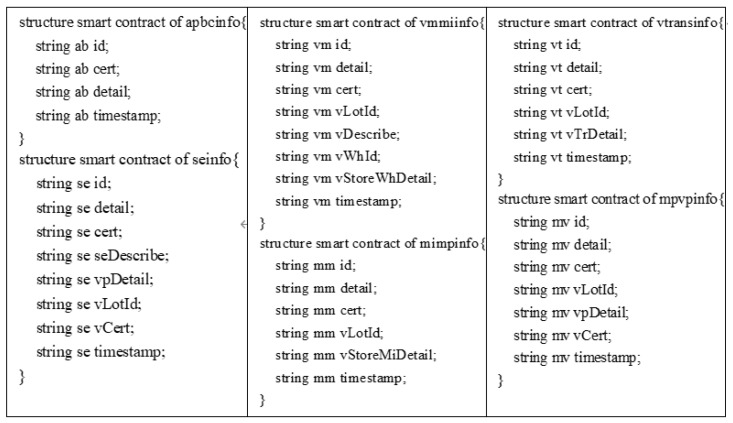
Smart contract structure of the scheme.

Moreover, the key information of each role designed within the system will be defined in the smart contract to ensure that the system can authenticate spontaneously and operate properly afterward. Figure 3 shows the smart contract framework associated with the system.

### 3.4. Registration Phase

**Figure 4:**
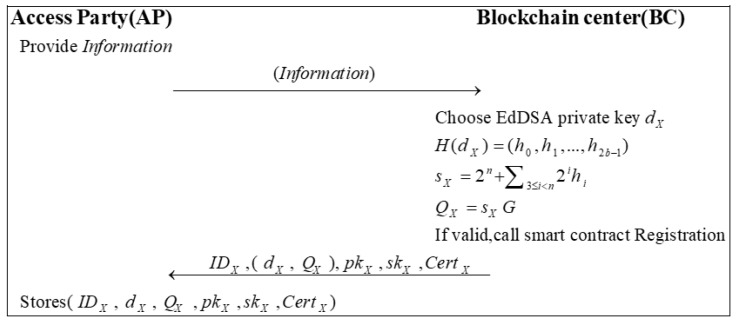
The process of the registration phase.

In this phase, the access parties (AP) in the system are registered through the blockchain center. After receiving the registration request, the blockchain center issues the roles with the corresponding public–private key pairs and a digital certificate that can prove their identity. Figure 4 displays the process of the registration phase.

Step 1: Each AP sends basic information about itself (e.g., role ID) to the BC.
Step 2: The BC uses the EdDSA algorithm to generate a private key $d_{X}$, then calculates $s_{X}$ and the corresponding public key $Q_{X}$ by the following:
  (1) $$H(d_{X})=(h_{0},h_{1},\ldots ,h_{2b-1})$$
  (2) $$s_{X}=2^{n}+\sum_{3\leq i<n}2^{i}h_{i}$$
  (3) $$Q_{X}=s_{X}G$$

If the identity of the AP is valid, the registered smart contract will be woken up. The algorithm for registration is shown in Algorithm 1. Then, the BC will send ${ID}_{X}$, $d_{X}$, $Q_{X}$, ${sk}_{X}$, ${pk}_{X}$, ${Cert}_{X}$ to AP.
**Algorithm 1:** The smart contract of registration.$\begin{array}{l} \mathrm{Var}\;\mathrm{APInfo}[]\;\mathrm{APs};\\ \mathrm{fuction}\;\mathrm{Registration}(\mathrm{String}\;x\_\mathrm{id},\;\mathrm{String}\;x\_\mathrm{detail},\;\mathrm{Roles}\;x\_\mathrm{roleType})\{\\ \;\;\mathrm{APInfo}\;\mathrm{ap}=\mathrm{new}\;\mathrm{APInfo}();\\ \;\;\mathrm{AP}.\mathrm{ID}=x\_\mathrm{id};\\ \;\;\mathrm{AP}.\mathrm{detail}=x\_\mathrm{detail};\\ \;\;\mathrm{AP}.\mathrm{roleType}=x\_\mathrm{roleType};\\ \;\;\mathrm{return}\;x\_\mathrm{keypairs};\\ \} \end{array}$

Step 3: The AP stores the $\left({ID}_{X},d_{X},Q_{X},{pk}_{X},{sk}_{X},{Cert}_{X},\right)$ for later signature and verification.

### 3.5. EdDSA Authentication Phase

**Figure 5:**
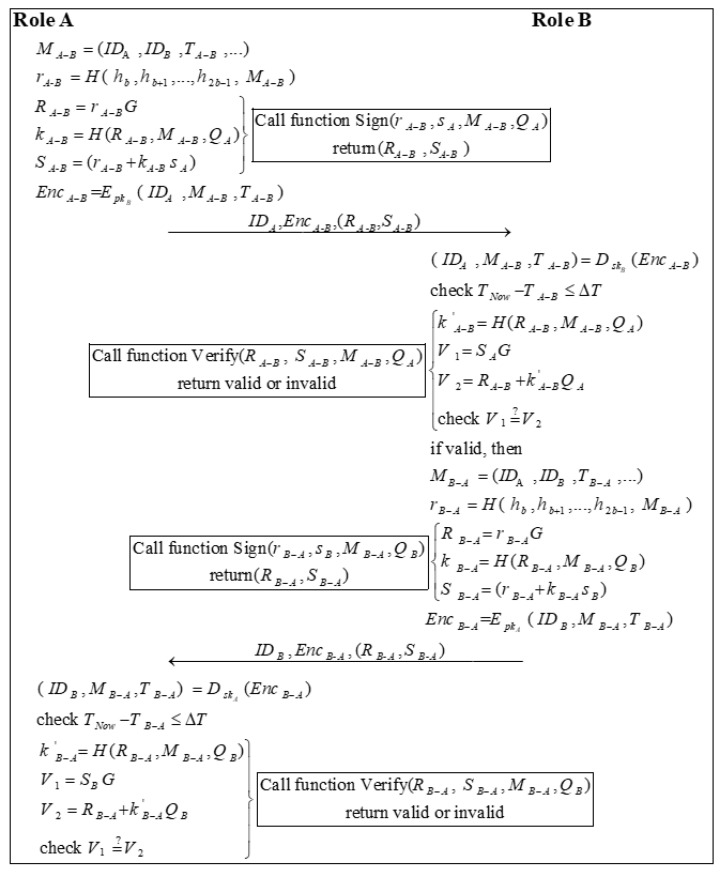
The process of the authentication phase.

Identity authentication is required before any two nodes communicate with each other. This phase is mainly in the form of mutual authentication of identity with the other node by using the EdDSA digital signature, and only two legitimate nodes can pass information between them. Role A and role B can represent the vaccine manufacturer (VM), the medical institution (MI), the medical person (MP), and the vaccinated person (VP). The process of the EdDSA Authentication Phase is shown in Figure 5. Algorithm 2 shows the signature process of EdDSA, and Algorithm 3 shows the verification process of EdDSA.

Step 1: Role A calculates a random number $r_{A-B}$ by encrypting the sent message $M_{A-B}$ with the high b bits of the hash of the private key:
  (4) rA−B=H( h,bh,b+1…,h,2b−1 M)A−B

Role A calls Algorithm 2 with (r,A−Bs,AM,A−BQ)A to sign the message and obtains the signature (R,A−BS)A−B. Subsequently, role A uses the public key of role B pkB to encrypt the message ( ID,AM,A−BT)A−B to generate EncA−B:

(5) Enc=A−BEpkB( ID,AM,A−BT)A−B

Role A sends ID,AEnc,A-B(R,A-BS)A-B to role B.
**Algorithm 2:** The process of the EdDSA signature between role A and role B.$\begin{array}{l} \mathrm{fuction}\;\mathrm{Sign}(\mathrm{String}\;r,\mathrm{String}\;s,\mathrm{String}\;M,\mathrm{String}\;Q)\{\\ \;\;R=r\times G;\\ \;\;k=H(R,M,Q);\\ \;\;S=(r+k\times s);\\ \;\;\mathrm{return}(R,S);\\ \} \end{array}$

Step 2: After receiving the message, role B first decrypts the message using its private key skB:
  (6) ( ID,AM,A−BT)A−B=DskB(Enc)A−B

Then, role B checks the validity of the timestamp:

(7) T−NowT≤A−BΔT

Next, role B calls Algorithm 3 to verify the signature based on the public information and the messages it received.

If the signature is valid, role B calculates a random number rB−A:

(8) r=B−AH( h,bh,b+1…,h,2b−1 M)B−A

Role B calls Algorithm 2 with (r,B−As,BM,B−AQ)B to sign the message and generates the signature (R,B−AS)B−A. Later, role B encrypts the message ( ID,BM,B−AT)B−A by using the public key of role A pkA to generate EncB−A:

(9) Enc=B−AEpkA( ID,BM,B−AT)B−A

Role B sends ID,BEnc,B-A(R,B-AS)B-A to role A.

Step 3: Firstly, role A decrypts the message EncB−A using its private key skA:
  (10) ( ID,BM,B−AT)B−A=DskA(Enc)B−A

Next, role A checks the validity of the timestamp:

(11) T−NowT≤B−AΔT

Finally, role A calls Algorithm 3 to verify the signature based on the public information and the received messages.
**Algorithm 3:** The process of EdDSA verification between role A and role B.fuction Verify(String R,String S,String M,String Q){  k=H(R,M,Q);  V=1S×G;  V=2R+k×Q;A  if V=1=V{2      return "valid" ;  }else{       return"invalid";}

### 3.6. Vaccine Purchasing Phase

**Figure 6:**
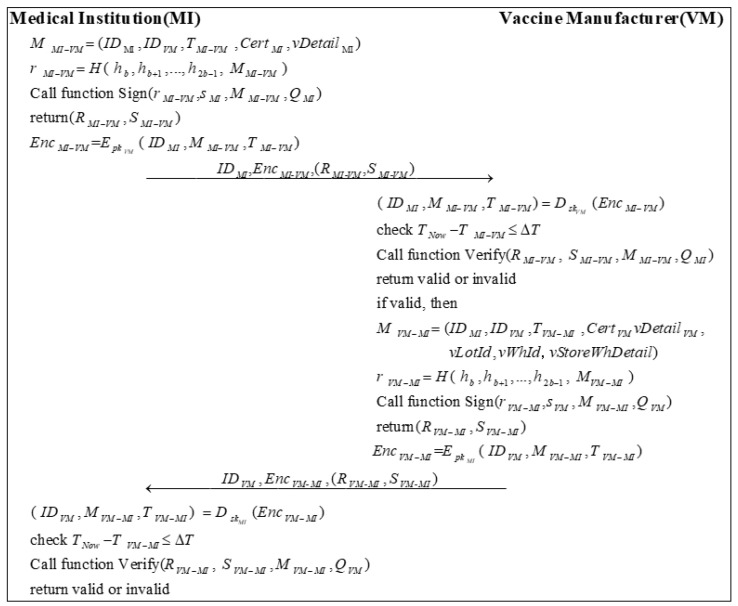
The process of vaccine purchase.

In the vaccine purchasing phase, the MI first issues vaccine purchase requests to the VM. The VM produces the vaccine according to the vaccine needs of the MI after confirming the identity of the MI. When the vaccine is made, the VM needs to submit the vaccine-related information to the BC. Figure 6 describes the process of the vaccine purchasing phase.

Step 1: MI sends a message MMI−VM to VM. MMI−VM needs to include the required vaccine details of the MI’s vDetailMI beside the primary information. Then, the MI calculates a random number rMI−VM by encrypting MMI−VM with the high b bits of the hash of the private key:
  (12) r=MI−VMH( h,bh,b+1…,h,2b−1 M)MI−VM

MI calls Algorithm 2 with (r,MI−VMs,MIM,MI−VMQ)MI to sign the message and obtains the signature (R,MI−VMS)MI−VM.

(13) (R,MI−VMS)MI−VM=Sign(r,MI−VMs,MIM,MI−VMQ)MI

Subsequently, MI uses the public key of the VM pkVM to encrypt the message ( ID,MIM,MI−VMT)MI−VM to generate EncMI−VM:

(14) Enc=MI−VMEpkVM( ID,MIM,MI−VMT)MI−VM

MI sends ID,MIEnc,MI-VM(R,MI-VMS)MI-VM to VM.

Step 2: After receiving the message, VM first decrypts the message EncMI−VM using its private key skVM:
  (15) ( ID,MIM,MI−VMT)MI−VM=DskVM(Enc)MI−VM

Then, VM checks the validity of the timestamp:

(16) T−NowT≤MI−VMΔT

Next, VM calls Algorithm 3 to verify the signature based on the public information and the messages it received.

(17) Verify(R,MI−VM S,MI−VMM,MI−VMQ)MI

If the signature is valid, VM sends a message MVM−MI to MI. Besides the basic information, MVM−MI needs to include the required vaccine details of the VM’s vDetailVM, the vaccine lot number vLotId, the vaccine storage warehouse number vWhId, and the vaccine storage warehouse details vStoreWhDetail. Then, VM calculates a random number rVM−MI:

(18) r=VM−MIH( h,bh,b+1…,h,2b−1 M)VM−MI

VM calls Algorithm 2 with (r,VM−MIs,VMM,VM−MIQ)VM to sign the message and generates the signature (R,VM−MIS)VM−MI.

(19) (R,VM−MIS)VM−MI=Sign(r,VM−MIs,VMM,VM−MIQ)VM

Later, VM encrypts the message ( ID,VMM,VM−MIT)VM−MI by using the public key of MI pkMI to generate EncVM−MI:

(20) Enc=VM−MIEpkMI( ID,VMM,VM−MIT)VM−MI

VM sends ID,VMEnc,VM-MI(R,VM-MIS)VM-MI to MI.

Step 3: First, MI decrypts the message EncVM−MI using its private key skMI:
  (21) ( ID,VMM,VM−MIT)VM−MI=DskMI(Enc)VM−MI

Next, MI checks the validity of the timestamp:

(22) T−NowT≤VM−MIΔT

Finally, MI calls Algorithm 3 to verify the signature based on the public information and the messages it receives.

(23) Verify(R,VM−MI S,VM−MIM,VM−MIQ)VM

### 3.7. Vaccine Transport Phase

**Figure 7:**
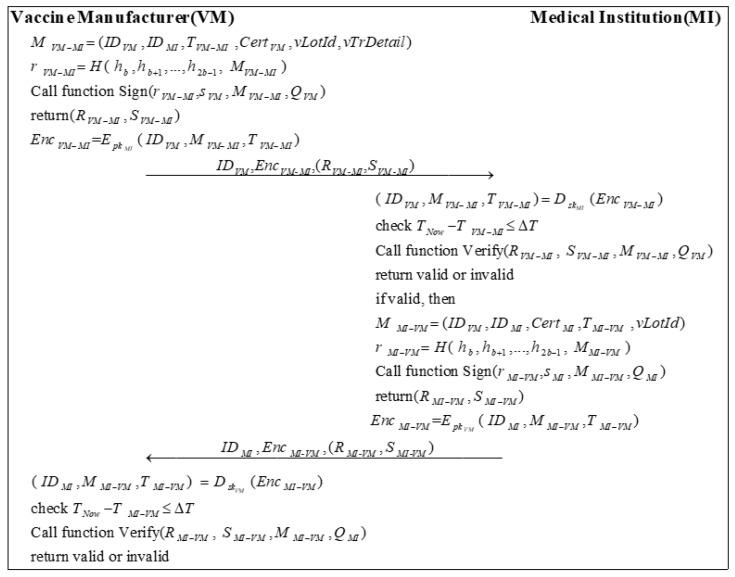
The process of vaccine transport.

In the previous phase, the VM completed the vaccine according to the requirements of the MI. Once the MI confirms that the vaccine information is correct, the system enters the vaccine transport phase. The information generated by the vaccine transport process will be updated in the BC. Finally, the MI sends a message to the VM to accept the vaccine after checking that the vaccine transport is in order. Figure 7 shows the process of the vaccine transport phase.

Step 1: VM sends a message MVM−MI to MI. MVM−MI needs to include the vaccine lot number vLotId, the vaccine transport details vTrDetail, and the primary information. Then, MI calculates a random number rVM−MI by encrypting MVM−MI with the high b bits of the hash of the private key:
  (24) r=VM−MIH( h,bh,b+1…,h,2b−1 M)VM−MI

VM calls Algorithm 2 with (r,MI−VMs,MIM,MI−VMQ)MI to sign the message and obtains the signature (R,VM−MIS)VM−MI.

(25) (R,VM−MIS)VM−MI=Sign(r,VM−MIs,VMM,VM−MIQ)VM

Subsequently, VM uses the public key of MI pkMI to encrypt the message ( ID,VMM,VM−MIT)VM−MI to generate EncVM−MI:

(26) Enc=VM−MIEpkMI( ID,VMM,VM−MIT)VM−MI

VM sends ID,MIEnc,MI-VM(R,MI-VMS)MI-VM to MI.

Step 2: After receiving the message, MI first decrypts the message EncVM−MI using its private key skMI:
  (27) ( ID,VMM,VM−MIT)VM−MI=DskMI(Enc)VM−MI

Then, MI checks the validity of the timestamp:

(28) T−NowT≤VM−MIΔT

Next, MI calls Algorithm 3 to verify the signature based on the public information and the messages it receives.

(29) Verify(R,VM−MI S,VM−MIM,VM−MIQ)VM

If the signature is valid, MI sends a message MMI−VM to VM. Besides the basic information, MMI−VM should include the vaccine lot number vLotId. Then, VM calculates a random number rMI−VM:

(30) r=MI−VMH( h,bh,b+1…,h,2b−1 M)MI−VM

MI calls Algorithm 2 with (r,MI−VMs,MIM,MI−VMQ)MI to sign the message and generates the signature (R,MI−VMS)MI−VM.

(31) (R,MI−VMS)MI−VM=Sign(r,MI−VMs,MIM,MI−VMQ)MI

Later, MI encrypts the message ( ID,MIM,MI−VMT)MI−VM by using the public key of the VM pkVM to generate EncMI−VM:

(32) Enc=MI−VMEpkVM( ID,MIM,MI−VMT)MI−VM

MI sends ID,MIEnc,MI-VM(R,MI-VMS)MI-VM to VM.

Step 3: First, VM decrypts the message EncMI−VM using its private key skVM:
  (33) ( ID,MIM,MI−VMT)MI−VM=DskVM(Enc)MI−VM

Next, VM checks the validity of the timestamp:

(34) T−NowT≤MI−VMΔT

Finally, VM calls Algorithm 3 to verify the signature based on the public information and the messages it received.

(35) Verify(R,MI−VM S,MI−VMM,MI−VMQ)MI

### 3.8. Vaccine Distributing Phase

**Figure 8:**
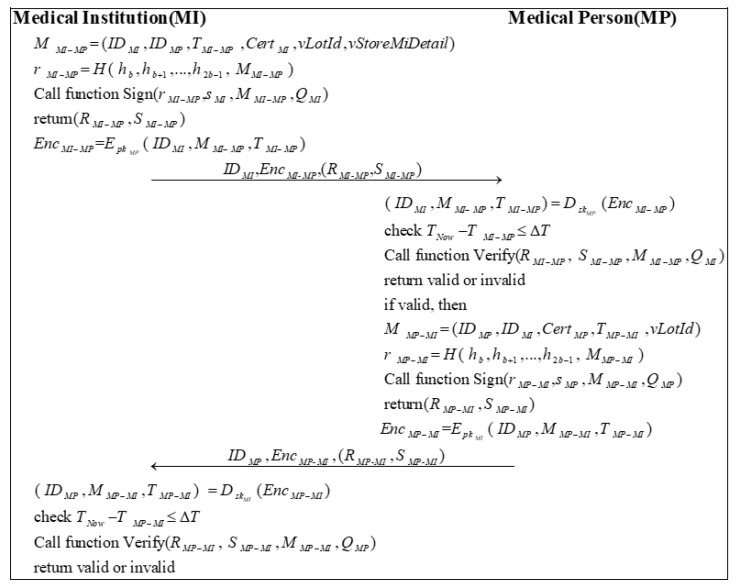
The process of vaccine distribution.

In this phase, the MI first needs to store the qualified vaccines received and manage them properly and strictly. Next, MI distributes the vaccines to the corresponding MP, based on the local government’s vaccination requirements. Figure 8 illustrates the entire process of the vaccine-distributing phase.

Step 1: MI sends a message MMI−MP to MP. MMI−MP needs to include the vaccine lot number vLotId, the vaccine storage details in MI vStoreMiDetail, and the primary information. Then, MI calculates a random number rMI−MP by encrypting MMI−MP with the high b bits of the hash of the private key:
  (36) r=MI−MPH( h,bh,b+1…,h,2b−1 M)MI−MP

MI calls Algorithm 2 with (r,MI−MPs,MIM,MI−MPQ)MI to sign the message and obtains the signature (R,MI−MPS)MI−MP.

(37) (R,MI−MPS)MI−MP=Sign(r,MI−MPs,MIM,MI−MPQ)MI

Subsequently, MI uses the public key of MP pkMP to encrypt the message ( ID,MIM,MI−MPT)MI−MP to generate EncMI−MP:

(38) Enc=MI−MPEpkMP( ID,MIM,MI−MPT)MI−MP

MI sends  ID,MIEnc,MI-MP(R,MI-MPS)MI-MP to MP.

Step 2: After receiving the message, MP first decrypts the message EncMI−MP using its private key skMP:
  (39) ( ID,MIM,MI−MPT)MI−MP=DskMP(Enc)MI−MP

Then, MP checks the validity of the timestamp:

(40) T−NowT≤MI−MPΔT

Next, MP calls Algorithm 3 to verify the signature based on the public information and the messages it receives.

(41) Verify(R,MI−MP S,MI−MPM,MI−MPQ)MI

If the signature is valid, MP sends a message MMP−MI to MI. Besides the basic information, MMP−MI should include the vaccine lot number vLotId. Then, MP calculates a random number  rMP−MI:

(42) r=MP−MIH( h,bh,b+1…,h,2b−1 M)MP−MI

MP calls Algorithm 2 with (r,MP−MIs,MPM,MP−MIQ)MP to sign the message and generates the signature (R,MP−MIS)MP−MI.

(43) (R,MP−MIS)MP−MI=Sign(r,MP−MIs,MPM,MP−MIQ)MP

Later, MP encrypts the message ( ID,MPM,MP−MIT)MP−MI by using the public key of MI pkMI to generate EncMP−MI:

(44) Enc=MP−MIEpkMI( ID,MPM,MP−MIT)MP−MI

MP sends ID,MPEnc,MP-MI(R,MP-MIS)MP-MI to MI.

Step 3: First, MI decrypts the message EncMP−MI using its private key skMI:
  (45) ( ID,MPM,MP−MIT)MP−MI=DskMI(Enc)MP−MI

Next, MI checks the validity of the timestamp:

(46) T−NowT≤MP−MIΔT

Finally, MI calls Algorithm 3 to verify the signature based on the public information and the messages it receives.

(47) Verify(R,MP−MI S,MP−MIM,MP−MIQ)MP

### 3.9. Vaccination Phase

**Figure 9:**
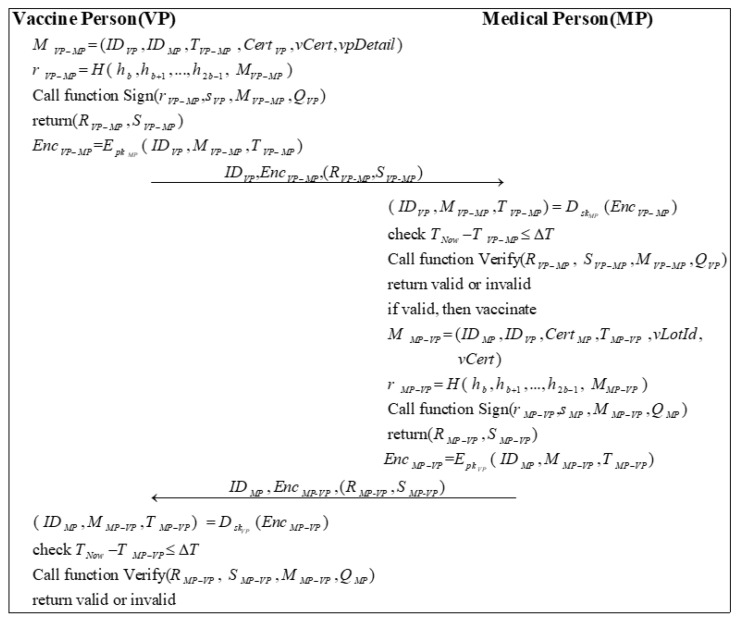
The process of vaccination.

This phase mainly involves vaccination of the VP. The VP must first submit personal information and vaccination status to the MP before vaccination. The vaccination is administered only after the MP confirms that the information is correct and that the VP is medically fit to receive the vaccine. Finally, the MP will also need to update the vaccination certificate of the vaccine recipient. Figure 9 shows the process of vaccination.

Step 1: VP sends a message MVP−MP to MP. MVP−MP needs to include the vaccination certificate of the VP vCert, the VP details vpDetail, and the primary information. Then, VP calculates a random number rVP−MP by encrypting MVP−MP with the high b bits of the hash of the private key:
  (48) r=VP−MPH( h,bh,b+1…,h,2b−1 M)VP−MP

VP calls Algorithm 2 with (r,VP−MPs,VPM,VP−MPQ)VP to sign the message and obtains the signature (R,VP−MPS)VP−MP.

(49) (R,VP−MPS)VP−MP=Sign(r,VP−MPs,VPM,VP−MPQ)VP

Subsequently, VP uses the public key of MP pkMP to encrypt the message ( ID,VPM,VP−MPT)VP−MP to generate EncVP−MP:

(50) Enc=VP−MPEpkMP( ID,VPM,VP−MPT)VP−MP

VP sends ID,VPEnc,VP−MP(R,VP-MPS)VP-MP to MP.

Step 2: After receiving the message, MP first decrypts the message EncVP−MP using its private key skMP:
  (51) ( ID,VPM,VP−MPT)VP−MP=DskMP(Enc)VP−MP

Then, MP checks the validity of the timestamp:

(52) T−NowT≤VP−MPΔT

Next, MP calls Algorithm 3 to verify the signature based on the public information and the messages it receives.

(53) Verify(R,VP−MP S,VP−MPM,VP−MPQ)VP

If the signature is valid, MP vaccinates VP. Afterward, MP sends a message MMP−VP to VP. Besides the basic information, MMP−VP should include the vaccine lot number vLotId and the vaccination certificate of the VP vCert. Then, MP calculates a random number rMP−VP:

(54) r=MP−VPH( h,bh,b+1…,h,2b−1 M)MP−VP

MP calls Algorithm 2 with (r,MP−VPs,MPM,MP−VPQ)MP to sign the message and generates the signature (R,MP−VPS)MP−VP.

(55) (R,MP−VPS)MP−VP=Sign(r,MP−VPs,MPM,MP−VPQ)MP

Later, MP encrypts the message ( ID,MPM,MP−VPT)MP−VP by using the public key of VP pkVP to generate EncMP−VP:

(56) Enc=MP−VPEpkVP( ID,MPM,MP−VPT)MP−VP

MP sends ID,MPEnc,MP-VP(R,MP-VPS)MP-VP to VP.

Step 3: First, VP decrypts the message EncMP−VP using its private key skVP:
  (57) ( ID,MPM,MP−VPT)MP−VP=DskVP(Enc)MP−VP

Next, VP checks the validity of the timestamp:

(58) T−NowT≤MP−VPΔT

Finally, VP calls Algorithm 3 to verify the signature based on the public information and the messages it received.

(59) Verify(R,MP−VP S,MP−VPM,MP−VPQ)MP

### 3.10. Side Effect Phase

**Figure 10:**
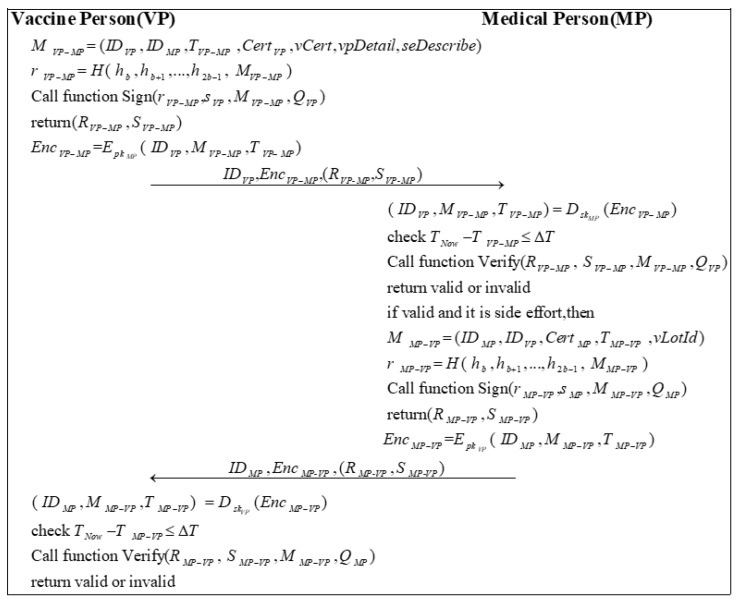
The process of the side effects submitted.

If a VP has an adverse reaction after receiving the vaccine, the VP is required to upload the appropriate information to the BC, including details of the adverse reaction, personal information, and proof of vaccination. If the adverse reaction is judged to be a side effect of the vaccination, the vaccine lot ID will be returned to the VP, and the appropriate treatment will be provided. Figure 10 shows the process of the side-effect phase.

Step 1: VP sends a message MVP−MP to MP. MVP−MP needs to include the vaccination certificate of VP vCert, the VP details vpDetail, the description of side effects seDescribe, and the primary information. Then, VP calculates a random number rVP−MP by encrypting MVP−MP with the high b bits of the hash of the private key:
  (60) r=VP−MPH( h,bh,b+1…,h,2b−1 M)VP−MP

VP calls Algorithm 2 with (r,VP−MPs,VPM,VP−MPQ)VP to sign the message and obtains the signature (R,VP−MPS)VP−MP.

(61) (R,VP−MPS)VP−MP=Sign(r,VP−MPs,VPM,VP−MPQ)VP

Subsequently, VP uses the public key of MP pkMP to encrypt the message ( ID,VPM,VP−MPT)VP−MP to generate EncVP−MP:

(62) Enc=VP−MPEpkMP( ID,VPM,VP−MPT)VP−MP

VP sends ID,VPEnc,VP−MP(R,VP-MPS)VP-MP to MP.

Step 2: After receiving the message, MP first decrypts the message EncVP−MP using its private key skMP:
  (63) ( ID,VPM,VP−MPT)VP−MP=DskMP(Enc)VP−MP

Then, MP checks the validity of the timestamp:

(64) T−NowT≤VP−MPΔT

Next, MP calls Algorithm 3 to verify the signature based on the public information and the messages it receives.

(65) Verify(R,VP−MP S,VP−MPM,VP−MPQ)VP

If the signature is valid and MP considers the adverse reaction to being a side effect of the vaccination, MP sends a message MMP−VP to VP. In addition to the basic information, MMP−VP should include the vaccine lot number. Then, MP calculates a random number rMP−VP:

(66) r=MP−VPH( h,bh,b+1…,h,2b−1 M)MP−VP

MP calls Algorithm 2 to sign the message (r,MP−VPs,MPM,MP−VPQ)MP and generates the signature (R,MP−VPS)MP−VP.

(67) (R,MP−VPS)MP−VP=Sign(r,MP−VPs,MPM,MP−VPQ)MP

Later, MP encrypts the message ( ID,MPM,MP−VPT)MP−VP by using the public key of VP pkVP to generate EncMP−VP:

(68) Enc=MP−VPEpkVP( ID,MPM,MP−VPT)MP−VP

MP sends ID,MPEnc,MP-VP(R,MP-VPS)MP-VP to VP.

Step 3: First, VP decrypts the message EncMP−VP using its private key skVP:
  (69) ( ID,MPM,MP−VPT)MP−VP=DskVP(Enc)MP−VP

Next, VP checks the validity of the timestamp:

(70) T−NowT≤MP−VPΔT

Finally, VP calls Algorithm 3 to verify the signature based on the public information and the messages it received.

(71) Verify(R,MP−VP S,MP−VPM,MP−VPQ)MP

## 4. Security Analysis

### 4.1. Mutual Authentication

The proposed scheme uses BAN logic to achieve mutual authentication between role A and role B. The scheme of role A and role B can represent the blockchain center (BC), the vaccine manufacturer (VM), the medical institution (MI), the medical personnel (MP), and the vaccinated person (VP). The notation of BAN logic is shown below.
P|≡XP believes X
P⊲XP sees X
P|∼XP said X
P|⇒XP controls X
#(X)The message X is fresh$P\overset{K}{↔}Q$P and Q communicate with a shared key K
${\left\{X\right\}}_{K}$X is encrypted with a key K


The goals of the entire authentication process are as follows:

$G1:A|\equiv A\overset{{}_{X_{A}}}{↔}B$

$G2:A|\equiv B|\equiv A\overset{{}_{X_{A}}}{↔}B$

$G3:B|\equiv A\overset{{}_{X_{B}}}{↔}B$

$G4:B|\equiv A|\equiv A\overset{{}_{X_{B}}}{↔}B$

$G5:A|\equiv ID_{B}$

$G6:A|\equiv B|\equiv ID_{B}$

$G7:B|\equiv ID_{A}$

$G8:B|\equiv A|\equiv ID_{A}$

Depending on the authentication process, BAN logic generates the following idealized model:

$M1:Role\;A\to Role\;B({\left\{ID_{A},ID_{B},T_{A-B}\right\}}_{PK_{B}},R_{A},R_{A})$

$M2:Role\;B\to Role\;A({\left\{ID_{A},ID_{B},T_{B-A}\right\}}_{PK_{A}},R_{B},R_{B})$

To analyze the proposed scheme, we make the following assumptions:

$A1:A|\equiv \#(T_{A-B})$

$A2:B|\equiv \#(T_{A-B})$

$A3:A|\equiv \#(T_{B-A})$

$A4:B|\equiv \#(T_{B-A})$

$A5:A|\equiv B|\Rightarrow B\overset{{}_{X_{B}}}{↔}A$

$A6:B|\equiv A|\Rightarrow A\overset{{}_{X_{A}}}{↔}B$

$A7:A|\equiv B|\Rightarrow ID_{B}$

$A8:B|\equiv A|\Rightarrow ID_{A}$

Based on the rules of BAN logic and the assumptions above, the authentication process between the two nodes is shown below:

- Role B authenticates role A.
  The statement S1 can be derived from M1 the seeing rule:
  $S1:B⊲({\left\{ID_{A},ID_{B},T_{A-B}\right\}}_{PK_{B}},R_{A},R_{A})$
  The statement S2 can be derived from A2 and the freshness rule:
  $S2:B|\equiv \#({\left\{ID_{A},ID_{B},T_{A-B}\right\}}_{PK_{B}},R_{A},R_{A})$
  The statement S3 can be derived from S1, A4 and the message meaning rule:
  $S3:B|\equiv A|∼(ID_{A},ID_{B},T_{A-B},R_{A},R_{A})$
  The statement S4 can be derived by S2, S3 and the nonce verification rule:
  $S4:B|\equiv A|\equiv (ID_{A},ID_{B},T_{A-B},R_{A},R_{A})$
  The statement S5 can be derived from S4 and the belief rule:
  $S5:B|\equiv A|\equiv A\overset{{}_{X_{A}}}{↔}B$
  The statement S6 can be derived from S5, A6 and the jurisdiction rule:
  $S6:B|\equiv A\overset{{}_{X_{A}}}{↔}B$
  The statement S7 can be derived from S4 and the belief rule:
  $S7:B|\equiv A|\equiv ID_{A}$
  The statement S8 can be derived from S7, A8 and the belief rule:
  $S8:B|\equiv ID_{A}$
- Role A authenticates role B.
  The statement S9 can be derived from M2 and the seeing rule:
  $S9:A⊲({\left\{ID_{A},ID_{B},T_{B-A}\right\}}_{PK_{A}},R_{B},R_{B})$
  The statement S10 can be derived from A1 and the freshness rule:
  $S10:A|\equiv \#({\left\{ID_{A},ID_{B},T_{B-A}\right\}}_{PK_{A}},R_{B},R_{B})$
  The statement S11 can be derived from S9, A3 and the message meaning rule:
  $S11:A|\equiv B|∼(ID_{A},ID_{B},T_{B-A},R_{B},R_{B})$
  The statement S12 can be derived by S10,S11 and the nonce verification rule:
  $S12:A|\equiv B|\equiv (ID_{A},ID_{B},T_{B-A},R_{B},R_{B})$
  The statement S13 can be derived from S12 and the belief rule:
  $S13:A|\equiv B|\equiv B\overset{{}_{X_{B}}}{↔}A$
  The statement S14 can be derived from S13,A5 and the jurisdiction rule:
  $S14:A|\equiv B\overset{{}_{X_{B}}}{↔}A$
  The statement S15 can be derived from S12 and the belief rule:
  $S15:A|\equiv B|\equiv ID_{B}$
  The statement S16 can be derived from S15,A7 and the belief rule:
  $S16:A|\equiv ID_{B}$

With Statement S6, S8, S14, S16, role A and role B can easily verify the identity of each other when passing messages.

### 4.2. Decentralization and Information Sharing

The essence of blockchain technology is a distributed ledger. In the proposed scheme, all registered nodes jointly maintain the entire vaccine information management system, and any information has to be uploaded to the chain through the consensus mechanism of the system. Meanwhile, the failure of a single node does not cause the whole system to break down. Moreover, the information uploaded to the blockchain requires the sender to use its private key for signature, and the information on the chain can be viewed by other registered nodes. These features not only ensure the safety and reliability of the uploaded information but also ensure the openness and transparency of this information and realize the trust relationship between unfamiliar nodes.

### 4.3. Traceable

Messages sent in a blockchain system should be accompanied by using Algorithm 2. This message, if proven to be valid, is permanently stored in the blockchain and cannot be tampered with. Therefore, other nodes in the blockchain can trace the message and guarantee the validity of the message by using Algorithm 3. In this way, the traceability of the system is achieved.

### 4.4. High-Quality Random Number

The security of digital signature algorithms, such as DSA and ECDSA, relies on high-quality random number generators to generate random numbers. Once the quality of the random numbers is not up to par, the information of the system users will also be compromised. The random number generation of the EdDSA algorithm is shown in Equations (4), (8), (12), (18), (24), (36), (42), (48), (54), (60) and (66).

The generation of a random number of EdDSAs relies on the user’s private key with the delivered message itself. This random number is naturally of high quality, which pretty much eliminates the problem of information leakage caused by the quality of the random number.

### 4.5. Integrity and Non-Repudiation

When two nodes communicate, they are very concerned about the integrity of the transmitted message. In our scheme, the EdDSA algorithm is used to generate the signature. The sender generates a specific signature when sending a message based on random numbers, message content, and other parameters. Any tampering with the parameters will change the original signature, and the original message cannot be inferred from the signature string. 

Meanwhile, the sender signs the message with its private key when sending it, and the receiver will use the sender’s public key to verify the signature when receiving the message. Therefore, the sender cannot deny the message it sent. 

The signature in Table 1 describes the data integrity proof for each stage, and the verification describes the non-repudiation proof for each stage.

### 4.6. Man-in-the-Middle Attacks

To avoid this potential risk, the proposed scheme requires the sender to encrypt the message with the public key of the receiver before sending it to the receiver. Therefore, only the receiver can decrypt the message with its private key and obtain the message content. Thus, it solves the problem of man-in-the-middle attacks that may exist in the system. The encryption process for the message is shown in Equations (5), (6), (9), (10), (14), (15), (20), (21), (26), (27), (32), (33), (38), (39), (44), (45), (50), (51), (56), (57), (62), (63), (68) and (69).

Scenario: The sender sends a message to the receiver. Before the message is delivered, the malicious attacker eavesdrops and modifies the message. 

Analysis: The sender encrypts the message with the receiver’s public key when sending the message. The malicious attacker does not have the receiver’s private key, so the attacker cannot decrypt the exact contents of the message.

### 4.7. Replay Attack

To avoid this potential risk, the proposed scheme requires a timestamp to be attached to the message when it is passed between users. Both the timestamp and the message content are encrypted by the sender using the public key of the receiver, so the timestamp can only be decrypted and obtained by the receiver using his private key. If a malicious attacker sends the same message to the receiver later, the system will compare the difference between the current time and the message timestamp. Then, if the difference is greater than the threshold, the message is illegal. Therefore, the problem of replay attacks is eliminated. The specific process is shown as follows:

(72) Enc=A−BEpkB( ID,AM,A−BT)A−B

(73) ( ID,AM,A−BT)A−B=DskB(Enc)A−B

(74) check T−NowT≤A−BΔT

Scenario: The malicious attacker sends an identical message to the receiver after listening to the message sent by the sender. 

Analysis: The receiver decrypts the message with its private key, obtains the corresponding timestamp, and compares the difference between the current time and the timestamp with the threshold. If the difference is greater than the queue value, the system determines that it is a replay attack and rejects the message.

### 4.8. Sybil Attack

To avoid this potential risk, blockchain can use the consensus mechanism to increase the entry barrier of nodes. At this point, the high cost makes the Sybil attack unrealistic because the malicious attacker must occupy more than half of the nodes of the whole system. In addition, the proposed scheme requires each user to obtain the corresponding ID number and EdDSA public and private key pairs in the registration phase, and all users entering the system must pass identity validation. The parameters related to the user’s identity are generated by the blockchain center using Equations (1)–(3). Then, the user stores these parameters.

(75) Stores( ID,X d,X Q,Xpk,Xsk,XCert)X

Scenario: The malicious nodes attempt to forge vast numbers of fake identities to access the blockchain system. 

Analysis: Every ID number is generated by the blockchain center with the corresponding and unique public and private key pairs and certificates. The attacker has no chance of obtaining the complete parameters, and its operations in the system are considered invalid. Thus, the Sybil attack is hardly successful.

## 5. Discussion

### 5.1. Computation Cost

In this section, we analyzed the performance of the system. Table 2 presents the performance analysis of each phase. We used asymmetrical encryption/decryption, hash functions, addition, subtraction, multiplication, and division operations as the basis for calculating the costs.

### 5.2. Comparison

In this section, we compare the proposed scheme with previous articles dealing with vaccine record security in Table 3.

### 5.3. Performance Analysis

In this section, we perform a performance analysis of the proposed scheme using a caliper, a blockchain performance testing framework that allows its users to test the system on different blockchain platforms and obtain the corresponding performance test results. All tests were run in the following environment: Intel(R) Core (TM) i7-7700HQ CPU @ 2.80GHz, 8GB RAM. We use Fabric 2.0.0 and Go 1.17.5. The operating system is Ubuntu 18.04.5 LTS. 

Due to the different communication protocols of the schemes in each article, it is hard to find suitable papers to compare their performances. Therefore, we only base our scheme on analyzing its performance. The performance of blockchain systems is usually evaluated in terms of throughput and latency. The throughput refers to the speed at which transactions are added to the ledger and represents the performance level of the system, which is expressed in transactions per second (TPS) at the testing time. Latency is an indicator of the time spent between the application initiating a transaction time and the received time. This is the first thing that users care about when using a blockchain system.

**Figure 11:**
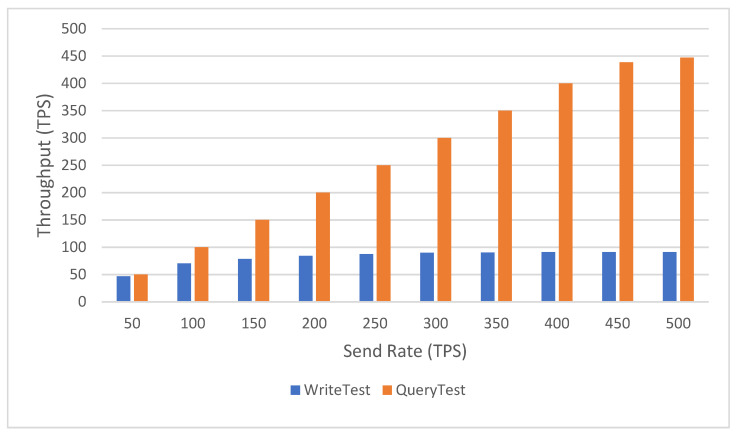
Throughput of the system at various send rates.

**Figure 12:**
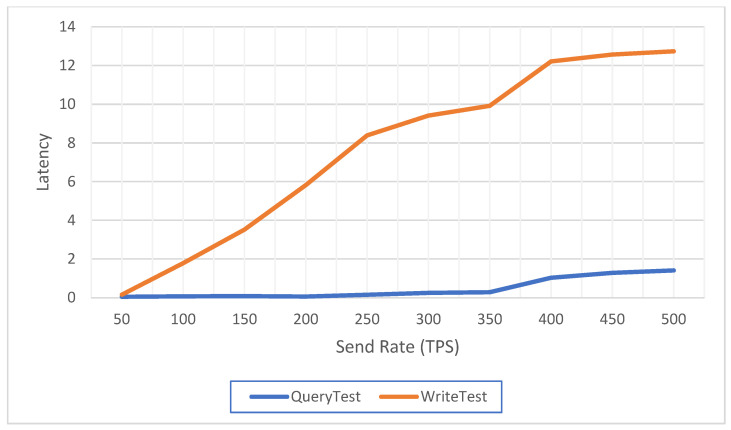
Latency of the system at various send rates.

Figure 11 shows the relationship between throughput and send rate. Ten sets of data were selected for the test, and the difference between the send rates of each set was 50 tps. With a fixed system block size, we can find that the throughput of read transactions is approximately linearly related to the send rate, ranging from a low of 50.1 tps to a high of 447.3 tps. The throughput of write transactions is positively correlated with the send rate, which grows gently from a low of 46.8 tps to a high of 91.2 tps. However, we can notice that the relative increase in throughput slows down after the send rate gradually increases, which could be approaching the threshold. Figure 12 shows the relationship between latency and the send rate. We can see that with fixed block size, latency is positively correlated with send rate, ranging from a minimum of 0.05 s to a maximum of 1.41 s for reading transactions, and from a minimum of 0.16 s to a maximum of 12.74 s for write transactions. Similarly, after the send rate reaches 400 tps, the latency growth rate becomes flatter, indicating that the system may have reached the threshold. Thus, the proposed system has adequate performance to read and write vaccine-related data. Meanwhile, users can access vaccine-related data in a short enough time and can modify these data according to their rights.

### 5.4. Comparison of Blockchain Platforms

Blockchain technology can be divided into three categories in total after development: public blockchain, private blockchain, and consortium blockchain. Private blockchain operation is centralized, and its node verification is usually operated by a single group only, which is not conducive to data sharing and traceability. Furthermore, the private blockchain is more susceptible to data tampering by unscrupulous elements, so they are not considered in this paper. Table 4 shows a comparison of the three blockchain platforms.

Compared to the public blockchain, consortium blockchain share information selectively, and nodes without permission cannot access the corresponding data. It can better protect the privacy of sensitive data, such as vaccine information. Moreover, Hyperledger Fabric has better performance and higher scalability. It also allows smart contracts to be written in multiple programming languages, making it easy to develop the system.

## 6. Conclusions

This paper proposes a vaccine record management system based on blockchain technology. The system protects the privacy of sensitive vaccine information while making the entire process of vaccine production, distribution, and vaccination transparent and traceable. It reduces the possibility of tampering with vaccine information and indirectly prevents vaccine quality failures.

Next, we analyzed the security of this vaccine information management system, including the use of BAN logic to achieve mutual authentication between communication nodes, and the EdDSA algorithm to ensure the integrity and non-repudiation of data. The EdDSA algorithm has the feature of selecting high-quality random numbers with excellent performance, which improves the efficiency of digital signatures and effectively avoids the security problems caused by low-quality random numbers that existed in some digital signature algorithms in the past. Furthermore, the proposed scheme can resist malicious attacks, such as man-in-the-middle attacks and replay attacks, to a certain extent.

In summary, this paper makes the following contributions:

1. Blockchain technology and smart contracts are used to ensure the security and traceability of vaccines from manufacturing, distribution, vaccination, and side-effect reporting. The system protects the privacy of each role while providing certain information about the vaccine based on the role’s identity.
2. The entire vaccine supply management system architecture and usage scenarios are presented.
3. The use of the EdDSA algorithm for digital signatures not only guarantees the integrity of vaccine-related records but also improves the security and efficiency of digital signatures.
4. Use BAN logic to guarantee mutual authentication between unfamiliar nodes.
5. Analyzed the potential security risks of the system.

## Figures and Tables

**Table 1:** Verification of integrity and non-repudiation in the proposed scheme.

Phase	Sender	Receiver	Signature	Verification
Authentication Phase	A	B	Sign(r,A−Bs,AM,A−BQ)A	Verify(R,A−B S,A−BM,A−BQ)A
	B	A	Sign(r,B−As,BM,B−AQ)B	Verify(R,B−A S,B−AM,B−AQ)B
Vaccine Purchasing Phase	MI	VM	Sign(r,MI−VMs,MIM,MI−VMQ)MI	Verify(R,MI−VM S,MI−VMM,MI−VMQ)MI
	VM	MI	Sign(r,VM−MIs,VMM,VM−MIQ)VM	Verify(R,VM−MI S,VM−MIM,VM−MIQ)VM
Vaccine transport Phase	VM	MI	Sign(r,VM−MIs,VMM,VM−MIQ)VM	Verify(R,MI−MP S,MI−MPM,MI−MPQ)MI
	MI	VM	Sign(r,MI−VMs,MIM,MI−VMQ)MI	Verify(R,MP−MI S,MP−MIM,MP−MIQ)MP
Vaccine Distributing Phase	MI	MP	Sign(r,MI−MPs,MIM,MI−MPQ)MI	Verify(R,MI−MP S,MI−MPM,MI−MPQ)MI
	MP	MI	Sign(r,MP−MIs,MPM,MP−MIQ)MP	Verify(R,MP−MI S,MP−MIM,MP−MIQ)MP
Vaccination Phase	VP	MP	Sign(r,VP−MPs,VPM,VP−MPQ)VP	Verify(R,VP−MP S,VP−MPM,VP−MPQ)VP
	MP	VP	Sign(r,MP−VPs,MPM,MP−VPQ)MP	Verify(R,MP−VP S,MP−VPM,MP−VPQ)MP
Side Effect Phase	VP	MP	Sign(r,VP−MPs,VPM,VP−MPQ)VP	Verify(R,VP−MP S,VP−MPM,VP−MPQ)VP
	MP	VP	Sign(r,MP−VPs,MPM,MP−VPQ)MP	Verify(R,MP−VP S,MP−VPM,MP−VPQ)MP

**Table 2:** The computation cost of each phase.

Phase	1st Party	2nd Party
Authentication Phase	Role A: $2T_{asy}+2T_{h}+2T_{add}+T_{sub}+4T_{mul}+2T_{com}$	Role B: $2T_{asy}+2T_{h}+2T_{add}+T_{sub}+4T_{mul}+2T_{com}$
Vaccine Purchasing Phase	$\mathrm{MI}:2T_{asy}+2T_{h}+2T_{add}+T_{sub}+4T_{mul}+2T_{com}$	$\mathrm{VM}:2T_{asy}+2T_{h}+2T_{add}+T_{sub}+4T_{mul}+2T_{com}$
Vaccine Transport Phase	$\mathrm{VM}:2T_{asy}+2T_{h}+2T_{add}+T_{sub}+4T_{mul}+2T_{com}$	$\mathrm{MI}:2T_{asy}+2T_{h}+2T_{add}+T_{sub}+4T_{mul}+2T_{com}$
Vaccine Distributing Phase	$\mathrm{MI}:2T_{asy}+2T_{h}+2T_{add}+T_{sub}+4T_{mul}+2T_{com}$	$\mathrm{MP}:2T_{asy}+2T_{h}+2T_{add}+T_{sub}+4T_{mul}+2T_{com}$
Vaccination Phase	$\mathrm{VP}:2T_{asy}+2T_{h}+2T_{add}+T_{sub}+4T_{mul}+2T_{com}$	$\mathrm{MP}:2T_{asy}+2T_{h}+2T_{add}+T_{sub}+4T_{mul}+2T_{com}$
Side Effect Phase	$\mathrm{VP}:2T_{asy}+2T_{h}+2T_{add}+T_{sub}+4T_{mul}+2T_{com}$	$\mathrm{MP}:2T_{asy}+2T_{h}+2T_{add}+T_{sub}+4T_{mul}+2T_{com}$

Notes: $T_{asy}$: asymmetrical encryption/decryption; $T_{h}$: a hash operation; $T_{add}$: an additional operation; $T_{sub}$: a subtraction operation. $T_{mul}$: a multiplication operation; $T_{com}$: the time required for a comparison operation.

**Table 3:** Comparison of the proposed and other vaccine-related articles.

Authors	Year	Objective	1	2	3	4	5	6
Sigwart et al. [27]	2019	Proposed the feasibility of blockchain application in the vaccine supply chain.	Y	N	Y	N	N	N
Yong et al. [28]	2019	Proposed to use of blockchain and machine learning to ensure vaccine safety.	Y	Y	Y	N	N	N
Deka et al. [30]	2020	Proposed to use of blockchain and IPFS to maintain personal vaccination records.	Y	Y	Y	N	N	N
Antal et al. [31]	2021	Proposed to use of smart contracts to monitor COVID-19 vaccine supply management.	Y	Y	N	N	N	Y
Chauhan et al. [32]	2021	Proposed to use of blockchain to ensure transparency and anti-counterfeiting of the COVID-19 vaccine.	Y	Y	N	N	N	Y
Chen et al. [33]	2022	Proposed a traceable blockchain-based vaccination record storage and share system.	Y	Y	Y	Y	Y	N
Our scheme	2022	Propose a blockchain-based traceable vaccine system.	Y	Y	Y	Y	Y	Y

Notes: 1: based on blockchain, 2: proposed system architecture, 3: used in multiple vaccines, 4: Mutual authentication, 5: security analysis, 6: Involved in the vaccine supply chain. Y: yes, N: no.

**Table 4:** Comparison of the three blockchain platforms.

Characteristics	Bitcoin	Ethereum	Hyperledger Fabric
Type	Public blockchain	Public blockchain	Consortium blockchain
Consensus	Proof of work (POW)	Proof of work (POW)	PBFT
Scripting	Limited stack-based scripting	Solidity	Go, Java, JavaScript
Authentication	No	No	Yes
Smart Contract	No	No	Yes
Scalability	Low scalability	Low scalability	High scalability
Currency	Bitcoin	Ether	No
Speed of transactions	7 TPS	20 TPS	1000 TPS-10000 TPS

## Data Availability

The data used to support the findings of this study are available from the corresponding author upon request.
